# Higher-than Expected Social Security Reliance Among Educated Black Americans: Minorities' Diminished Returns in National Health Interview Survey (NHIS) 2023

**DOI:** 10.31586/jbls.2025.1145

**Published:** 2025-02-10

**Authors:** Shervin Assari, Amanda Sonnega, Babak Najand, Hossein Zare

**Affiliations:** 1 Department of Internal Medicine, Charles R. Drew University of Medicine and Science, Los Angeles, CA, United States; 2 Department of Family Medicine, Charles R. Drew University of Medicine and Science, Los Angeles, CA, United States; 3 Department of Urban Public Health, Charles R. Drew University of Medicine and Science, Los Angeles, CA, United States; 4 Marginalization-Related Diminished Returns (MDRs) Center, Los Angeles, CA, United States; 5 Institute for Social Research, University of Michigan, Ann Arbor, MI, United States; 6 Department of Health Policy and Management, Johns Hopkins Bloomberg School of Public Health, Baltimore, MD, United States; 7 School of Business, University of Maryland Global Campus (UMGC), Adelphi, United States

**Keywords:** Education, Social Determinants, Population Groups

## Abstract

**Background:**

While educational attainment is generally associated with reduced reliance on Social Security and disability benefits, Minorities' Diminished Returns (MDRs) theory suggests that the socioeconomic benefits of educational attainment are not equally distributed across racial groups and are weaker for minoritized populations. This study explores the association between educational attainment and reliance on Social Security and disability benefits among Black and White adults in the United States.

**Objective:**

Building on the MDRs framework, we analyzed data from the National Health Interview Survey (NHIS) 2023 to examine how educational attainment impacts reliance on Social Security disability income, disability benefits, and public assistance for Black and White adults.

**Methods:**

We used a nationally representative sample of Black and White adults from the NHIS 2023 dataset. The outcomes assessed were reliance on three income sources: (1) Social Security disability income, (2) disability benefit income, and (3) public assistance disability income. Educational attainment was classified into three levels: less than high school (reference), high school diploma to some college, and college graduate or more. Logistic regression models assessed the relationship between educational attainment and reliance on each income source, with separate analyses for Black and White adults to evaluate differential effects.

**Results:**

Higher levels of educational attainment (high school diploma to some college and college graduate or more) were associated with lower odds of relying on Social Security disability, disability benefits, and public assistance. However, the protective effects of educational attainment were notably stronger for White adults than for Black adults. Among Black adults, even high educational attainment showed limited effectiveness in reducing reliance on these income sources, underscoring the Minorities' Diminished Returns (MDRs) phenomenon.

**Conclusions:**

Although educational attainment reduces reliance on Social Security and disability-related income sources, these protective effects are less pronounced for Black adults compared to White adults. The findings reveal persistent racial disparities in the economic returns of education, suggesting that structural factors may undermine the socioeconomic and health benefits of educational achievement for Black Americans. Targeted policy interventions may be needed to improve economic stability for Black adults, including those with higher educational credentials.

## 1. Introduction

Economic and health disparities between Black and White Americans remain persistent issues in the United States, with Black Americans experiencing poorer health and economic outcomes across most indicators, including life expectancy, rates of chronic illness, and financial stability [1-5]. Traditional approaches to understanding these disparities have largely focused on differential exposure to risk factors, such as poverty, discrimination, and stress, which disproportionately affect Black Americans [6,7]. However, emerging evidence suggests an additional mechanism: due to systemic racism and social stratification, Black Americans experience reduced economic and health benefits from equivalent levels of socioeconomic and psychological resources compared to White Americans. This phenomenon, known as Minorities' Diminished Returns (MDRs) [8,9], underscores the importance of not only providing access to resources but also addressing the differential effectiveness of these resources across racialized and minoritized groups.

The MDRs literature [10-18] suggests that even when Black individuals achieve higher levels of education, income, or social status, the resulting economic and health benefits are consistently smaller than those observed among their White counterparts. Several mechanisms have been proposed to explain these diminished returns, each rooted in structural inequities and historical biases. One such mechanism involves labor market preferences and practices, where Black Americans, despite similar educational attainment, are disproportionately represented in low-paying, high-stress jobs. Systemic discrimination within the labor market restricts career advancement and wage growth, thereby diminishing the benefits typically associated with stable, well-compensated employment. Discrimination, both interpersonal and institutional, also places a substantial psychological and physiological burden on Black individuals. Chronic exposure to discrimination is linked to increased rates of mental health issues, such as depression and anxiety, as well as physical health conditions, including hypertension and cardiovascular disease. Such discrimination can undermine the benefits of educational attainment, income, and other resources, particularly when Black Americans encounter bias within healthcare, educational, and financial systems [8].

Building on the MDRs framework [8,9], this study analyzed data from the National Health Interview Survey (NHIS) 2023 [19,20] to examine how educational attainment impacts reliance on Social Security disability income, disability benefits, and public assistance among Black and White adults. Using a nationally representative sample of Black and White adults from the NHIS 2023 dataset [21,22], we assessed reliance on three income sources: (1) Social Security disability income, (2) disability benefit income, and (3) public assistance disability income [23-25]. This analysis aims to deepen understanding of MDRs in economic outcomes, specifically within the context of public assistance reliance.

## 2. Methods

### 2.1. National Health Interview Survey (NHIS)

The National Health Interview Survey (NHIS) is the principal source of health data for the civilian, noninstitutionalized U.S. population, conducted by the National Center for Health Statistics (NCHS) since 1957. Authorized by the National Health Survey Act of 1956, NHIS continuously gathers data on health and disability across various demographics and socioeconomic groups, supporting research and policy initiatives within the Department of Health and Human Services and beyond [22].

### 2.2. NHIS 2023

In 2023, the NHIS conducted 29,522 Sample Adult interviews and 7,692 Sample Child interviews, achieving response rates of 47.0% and 44.9%, respectively. Data were collected through both in-person and telephone interviews, with 54.5% of interviews conducted at least partially by phone, similar to 2022 but higher than pre-pandemic levels in 2019 [26].

### 2.3. Design

NHIS is a cross-sectional, continuous survey covering the U.S. civilian noninstitutionalized population in the 50 states and the District of Columbia. Data are collected throughout the year, allowing monthly samples to be nationally representative. To manage costs and logistics, NHIS employs a geographically clustered sampling design [26].

### 2.4. Sampling

The sampling process begins by partitioning the United States into 1,689 geographic areas, based on counties or groups of counties. These areas are further stratified by population density in some states, while smaller states and the District of Columbia remain unstratified. Clusters within each stratum are selected proportionally, ensuring a representative national sample [26].

### 2.5. Sample

The NHIS targets individuals in households and noninstitutional group quarters, such as homeless shelters and group homes, but excludes active-duty military personnel, persons in long-term care institutions, and residents in correctional facilities [26].

### 2.6. Process and Interview

The U.S. Census Bureau conducts NHIS interviews under contract, deploying approximately 864 trained interviewers nationwide. Interviewers use computer-assisted personal interviewing (CAPI) technology, which facilitates question routing, real-time data entry, and data validation. Respondents receive an advance letter explaining NHIS participation, ensuring informed, voluntary consent. Interviews are conducted primarily in person, with follow-ups or special requests handled by phone [26].

### 2.7. Analytical Sample for this Paper

This paper used a sample of 22,746 participants, representative of 190,412,768 White and Black adults in the U.S. Eligibility criteria included having data on race/ethnicity, identifying as non-Latino White or non-Latino Black, and being an adult. Individuals of any other race, as well as all Latino or Hispanic individuals, were excluded from the analysis.

### 2.8. Measures

#### Educational Attainment:

Participants reported their highest level of education as one of the following categories: 00 (Never attended/kindergarten only), 01 (Grade 1-11), 02 (12th grade, no diploma), 03 (GED or equivalent), 04 (High school graduate), 05 (Some college, no degree), 06 (Associate degree: occupational, technical, or vocational program), 07 (Associate degree: academic program), 08 (Bachelor’s degree, e.g., BA, BS, BBA), 09 (Master’s degree, e.g., MA, MS, MBA), and 10 (Professional or doctoral degree, e.g., MD, JD, PhD). For analysis, we consolidated these categories into three groups: (1) Less than high school diploma (categories 0-3), (2) Some college (categories 4-7), and (3) College degree or higher (categories 8+). Educational attainment was treated as a three-level categorical variable, with "less than high school diploma" as the reference category, and "some college" and "college degree or higher" as the other two levels compared to this reference.

### 2.9. Outcomes

#### Social Security Income (No=0, Yes=1):

All participants were asked if they receive income from Social Security or Railroad Retirement, with response options of "yes," "no," or "refused to answer." This variable was coded as a binary outcome: 0 for "no" and 1 for "yes."

#### Social Security Disability Income (No=0, Yes=1):

All participants were asked if they receive Supplemental Security Income (SSI) or Social Security Disability Income (SSDI), distinguishing it from standard Social Security. Responses included "yes," "no," or "refused to answer." This variable was coded as a binary outcome: 0 for "no" and 1 for "yes."

#### Disability Benefit Income (No=0, Yes=1):

The subset of the participants who indicated receipt of SSI or SSDI were further asked if this income was specifically received as a disability benefit. Possible responses were "yes," "no," or "refused to answer." This variable was coded as a binary outcome: 0 for "no" and 1 for "yes."

#### Income from Public Assistance (No=0, Yes=1):

All participants were asked if they receive any public assistance or welfare payments from a state or local welfare office, with options of "yes," "no," or "refused to answer." This variable was coded as a binary outcome: 0 for "no" and 1 for "yes."

### 2.10. Covariates

Gender (female=0, male =1), age (years), marital status (other =0, married =1), last week employment status (other = 0, employed =1) . All covariates were self-report.

### 2.11. Ethics

This study was conducted in compliance with ethical standards, ensuring the protection and confidentiality of participant information. All data were collected anonymously, and no identifying information was retained. The study adhered to the ethical principles outlined in the Declaration of Helsinki, emphasizing respect, beneficence, and justice in research practices. Written informed consent was obtained from all participants prior to their involvement in the study, following a clear explanation of the study’s purpose, procedures, potential risks, and benefits. Institutional Review Board (IRB) approval was obtained for NHIS to ensure that all ethical guidelines were rigorously followed throughout the research process. Current analysis used fully deidentified existing data and did not need a full IRB review.

### 2.12. Statistical Analysis

We conducted all analyses using Stata, accounting for the survey design variables, including survey weights and strata, to ensure accurate representation of the non-Latino White and non-Latino Black U.S. adult populations. Given our focus on these groups exclusively, we applied subpopulation logistic regression techniques. With four outcomes of interest, we initially ran four separate logistic regression models without interaction terms. These models tested the additive effects of race and education on each outcome, adjusting for age, gender, marital status, and employment status as covariates. As all participants were non-Latino, ethnicity was not included as a control variable. Next, we conducted a second set of four logistic regression models, this time including interaction terms between race and education. These models retained all covariates from the initial models, allowing us to assess whether the effect of education on each outcome differed between non-Latino White and non-Latino Black adults. From each logistic regression model, we reported the odds ratios (OR), standard errors (SE), 95% confidence intervals (CI), and p-values. Our results consistently showed that higher educational attainment was inversely associated with reliance on Social Security and disability-related income (odds ratios less than one), indicating a protective effect of education. However, the race-by-education interaction terms revealed that this protective effect was significantly weaker for Black adults compared to White adults, as evidenced by interaction odds ratios greater than one. This suggests a diminished return on education for Black Americans in terms of reducing reliance on Social Security and disability income sources. All results presented are representative of non-Latino White and Black American adults.

## 3. Results

### 3.1. Descriptive Results

Table 1 presents descriptive statistics for the study sample, categorized by race, education level, marital status, employment status, gender, and various income support measures. The sample comprises primarily White participants (83.5%, SE = 0.58), with Black participants accounting for 16.5% (SE = 0.58).

Educational attainment levels show that 9.4% (SE = 0.31) of participants have less than a high school diploma, 55.9% (SE = 0.46) are high school graduates, and 34.6% (SE = 0.51) have a bachelor’s degree or higher. Marital status is nearly evenly split, with 50.3% (SE = 0.46) not married and 49.7% (SE = 0.46) married.

Regarding employment in the previous week, 43.2% (SE = 0.44) reported being unemployed, while 56.8% (SE = 0.44) were employed. Gender distribution is also balanced, with females making up 50.7% (SE = 0.40) and males 49.3% (SE = 0.40).

Income support reliance varies across categories. For Social Security income, 30.4% (SE = 0.42) of participants receive this income, while 69.6% (SE = 0.42) do not. Social Security Disability Income (SSDI) is received by 9.5% (SE = 0.28) of participants, with 90.5% (SE = 0.28) not receiving it. Disability Benefit Income is reported by 8.1% (SE = 0.25), while 91.9% (SE = 0.25) do not rely on it. Income from public assistance is the least common, with only 3.1% (SE = 0.18) of participants receiving it and 96.9% (SE = 0.18) not receiving this type of assistance.

As Table 1 shows, 8.5% (SE = 0.30) of White participants have less than a high school diploma, compared to 14.2% (SE = 0.90) of Black participants. High school graduates make up 54.7% (SE = 0.50) of the White sample and 62.1% (SE = 1.05) of the Black sample. A higher proportion of White participants have a bachelor’s degree or more (36.8%, SE = 0.56) compared to Black participants (23.7%, SE = 0.93). For marital status, 53.4% (SE = 0.48) of White participants are married, whereas only 30.8% (SE = 1.04) of Black participants are married. Conversely, a larger proportion of Black participants (69.2%, SE = 1.04) are in the “other” marital status category compared to White participants (46.6%, SE = 0.48). Regarding employment in the past week, the distribution is similar between groups, with 56.8% (SE = 0.47) of White participants and 56.6% (SE = 1.12) of Black participants reporting employment. Unemployment rates are nearly identical, with 43.2% (SE = 0.47) for Whites and 43.4% (SE = 1.12) for Blacks.

The mean age of White participants is 50.6 years (SE = 0.20), while the mean age of Black participants is 46.1 years (SE = 0.40), indicating that the White sample is, on average, slightly older than the Black sample. For gender, 50.1% (SE = 0.42) of White participants are female, compared to 53.5% (SE = 1.05) of Black participants. Male participants constitute 49.9% (SE = 0.42) of the White sample and 46.5% (SE = 1.05) of the Black sample.

In terms of Social Security income, 31.4% (SE = 0.46) of White participants receive Social Security income, compared to 24.9% (SE = 0.97) of Black participants. For Social Security Disability Income (SSDI), 8.5% (SE = 0.29) of White participants and 14.3% (SE = 0.84) of Black participants receive SSDI, indicating a higher reliance on disability income among Black participants. Disability Benefit Income shows similar patterns, with 7.3% (SE = 0.25) of White participants and 12.3% (SE = 0.82) of Black participants receiving this income, again indicating higher rates among Black participants. For income from public assistance, 2.6% (SE = 0.16) of White participants receive public assistance, compared to 5.6% (SE = 0.62) of Black participants.

### 3.2. Models 1 (Without Interaction)

Table 2 presents the results of logistic regression analyses examining the association between demographic, socioeconomic, and employment factors with reliance on different types of income support, including Social Security income, Social Security disability income, disability benefit income, and income from public assistance. Each regression was conducted without interaction terms.

For Social Security income, higher educational attainment was linked to lower odds of receiving Social Security income, with those holding a college degree having a 34% reduction in odds compared to individuals with less than a high school diploma (OR = 0.66, 95% CI [0.55, 0.78], p < 0.001). Family structure, specifically being married, was associated with an 18% reduction in the odds of receiving Social Security income (OR = 0.82, 95% CI [0.75, 0.90], p < 0.001). Employment during the previous week significantly decreased the odds of receiving Social Security income by 64% (OR = 0.36, 95% CI [0.32, 0.39], p < 0.001). Black and White individuals did not differ in their odds of receiving Social Security income (OR = 0.89, 95% CI [0.76, 1.04], p = 0.134). Age was positively associated with Social Security income, with each additional year of age increasing the odds by 9% (OR = 1.09, 95% CI [1.09, 1.10], p < 0.001). Gender did not significantly affect the odds of receiving Social Security income, as males had an odds ratio of 0.99 (95% CI [0.90, 1.08], p = 0.757).

For Social Security disability income, educational attainment significantly reduced the odds of receiving Social Security disability income, with individuals holding a college degree showing an 83% reduction in odds (OR = 0.17, 95% CI [0.14, 0.21], p < 0.001). Black individuals had significantly higher odds of receiving disability income compared to White individuals (OR = 1.47, 95% CI [1.25, 1.73], p < 0.001). Employment during the past week was associated with a 67% reduction in the odds of receiving Social Security disability income (OR = 0.33, 95% CI [0.28, 0.38], p < 0.001). Being married was associated with a 22% reduction in the odds of receiving disability income (OR = 0.78, 95% CI [0.68, 0.88], p < 0.001). Age had a minor but statistically significant effect (OR = 1.00, 95% CI [0.99, 1.00], p = 0.026). Males were 19% more likely to receive Social Security disability income than females (OR = 1.19, 95% CI [1.05, 1.34], p = 0.005).

For disability benefit income, educational attainment was strongly associated with lower odds of receiving disability benefits, with college graduates experiencing an 86% reduction in odds (OR = 0.14, 95% CI [0.12, 0.18], p < 0.001). Black individuals had 44% higher odds of receiving disability benefits compared to White individuals (OR = 1.44, 95% CI [1.21, 1.71], p < 0.001). Being married was linked to a 15% reduction in the odds of receiving disability benefits (OR = 0.85, 95% CI [0.74, 0.96], p = 0.012). Employment was associated with a 69% reduction in the odds of receiving disability benefit income (OR = 0.31, 95% CI [0.26, 0.36], p < 0.001). Each additional year of age was associated with a slight reduction in the odds of receiving disability benefit income (OR = 0.99, 95% CI [0.99, 1.00], p < 0.001). Males were 22% more likely to receive disability benefits than females (OR = 1.22, 95% CI [1.08, 1.39], p = 0.002).

For income from public assistance, higher education was significantly associated with lower odds of receiving public assistance income; those with a college degree had a 75% reduction in odds (OR = 0.25, 95% CI [0.18, 0.35], p < 0.001). Black individuals had 67% higher odds of receiving public assistance income compared to White individuals (OR = 1.67, 95% CI [1.28, 2.19], p < 0.001). Being married was associated with a 29% reduction in the odds of receiving public assistance income (OR = 0.71, 95% CI [0.55, 0.91], p = 0.007). Employment during the past week was linked to a 43% reduction in the odds of receiving income from public assistance (OR = 0.57, 95% CI [0.46, 0.71], p < 0.001). Age was inversely associated with public assistance income, with each additional year of age decreasing the odds (OR = 0.98, 95% CI [0.98, 0.99], p < 0.001). Males had 36% lower odds of receiving public assistance income than females (OR = 0.64, 95% CI [0.53, 0.78], p < 0.001).

### 3.3. Models 2 (With Interaction)

Table 3 presents the results of logistic regression analyses assessing the relationship between demographic, socioeconomic, and employment factors with reliance on various types of income support. These analyses include interaction terms between race and educational attainment, exploring how educational benefits on income reliance differ for Black and White adults.

For Social Security income, higher educational attainment was associated with a reduced likelihood of receiving Social Security income; individuals with a college degree had a 42% reduction in odds compared to those with less than a high school diploma (OR = 0.58, 95% CI [0.47, 0.70], p < 0.001). Notably, the interaction terms showed that Black individuals with a high school diploma or college degree experienced significantly higher odds of receiving Social Security income than their White counterparts, with interaction odds ratios of 1.79 (95% CI [1.16, 2.74], p = 0.008) and 1.75 (95% CI [1.09, 2.81], p = 0.021), respectively. Age was positively associated with Social Security income, with each additional year of age increasing the odds by 9% (OR = 1.09, 95% CI [1.09, 1.10], p < 0.001). Gender had no significant effect (OR = 0.99, 95% CI [0.90, 1.08], p = 0.761). Being married reduced the odds of receiving Social Security income by 18% (OR = 0.82, 95% CI [0.75, 0.90], p < 0.001), and employment during the past week reduced the odds by 64% (OR = 0.36, 95% CI [0.32, 0.39], p < 0.001).

For Social Security disability income, education showed a strong protective effect, with a college degree reducing the odds by 85% (OR = 0.15, 95% CI [0.12, 0.19], p < 0.001). Interaction terms indicated that Black individuals with a college degree had higher odds of receiving Social Security disability income compared to their White counterparts, although this interaction effect approached but did not reach significance (OR = 1.64, 95% CI [0.99, 2.70], p = 0.053). Age was associated with a slight but significant effect (OR = 1.00, 95% CI [0.99, 1.00], p = 0.028). Males had higher odds of receiving Social Security disability income (OR = 1.19, 95% CI [1.06, 1.34], p = 0.004), while being married was linked to a 22% reduction in odds (OR = 0.78, 95% CI [0.69, 0.88], p < 0.001). Employment decreased the odds by 67% (OR = 0.33, 95% CI [0.28, 0.38], p < 0.001).

For disability benefit income, higher education significantly reduced the odds, with a college degree linked to an 87% reduction (OR = 0.13, 95% CI [0.11, 0.17], p < 0.001). The interaction terms for education and race were not significant, indicating that the protective effect of education on disability benefit reliance did not differ by race (p > 0.05). Age had a slight protective effect (OR = 0.99, 95% CI [0.99, 1.00], p < 0.001), and males had higher odds of receiving disability benefits (OR = 1.23, 95% CI [1.08, 1.39], p = 0.002). Being married was associated with a 15% reduction in the odds of receiving disability benefits (OR = 0.85, 95% CI [0.74, 0.97], p = 0.014), and employment was associated with a 69% reduction in odds (OR = 0.31, 95% CI [0.26, 0.36], p < 0.001).

For income from public assistance, educational attainment provided strong protective effects, with a college degree reducing the odds of public assistance reliance by 81% (OR = 0.19, 95% CI [0.13, 0.28], p < 0.001). The interaction effect between race and a college degree was significant, indicating that Black individuals with a college degree had higher odds of relying on public assistance income than their White counterparts (OR = 2.69, 95% CI [1.27, 5.70], p = 0.010). Age was negatively associated with public assistance income, with each additional year of age decreasing the odds (OR = 0.98, 95% CI [0.98, 0.99], p < 0.001). Males had 36% lower odds of receiving public assistance income compared to females (OR = 0.64, 95% CI [0.53, 0.78], p < 0.001). Being married reduced the odds by 28% (OR = 0.72, 95% CI [0.56, 0.92], p = 0.009), and employment was associated with a 43% reduction in odds (OR = 0.57, 95% CI [0.46, 0.70], p < 0.001).

Overall, these results reveal that while higher education generally reduces reliance on income support, Black individuals, particularly those with higher education, may not experience these benefits to the same extent as White individuals. This finding aligns with the Minorities' Diminished Returns framework, highlighting racial disparities in the economic returns on educational attainment.

## 4. Discussion

This study built on the Minorities' Diminished Returns (MDRs) framework to examine whether educational attainment impacts reliance on Social Security disability income, disability benefits, and public assistance among Black and White adults in the United States. We hypothesized that while higher levels of educational attainment would generally reduce reliance on these sources of income, the protective effects of education would be weaker for Black adults than for White adults, reflecting MDRs in economic and health outcomes.

Our findings support the MDRs framework [27-38], showing that higher educational attainment was associated with reduced odds of relying on Social Security disability income, disability benefits, and public assistance. However, the protective effects of education were notably weaker among Black adults compared to their White counterparts. Despite increased educational credentials, Black adults showed fewer reductions in reliance on these income sources, underscoring how structural barriers may undermine the socioeconomic and health benefits of educational attainment for Black populations.

Extensive research indicates that higher educational attainment is generally associated with improved economic stability and health outcomes, including better job opportunities, higher income, and reduced reliance on public assistance programs [39-43]. Education also lowers the odds of poverty and dependence on Social Security. Our findings align with previous studies on MDRs, which demonstrate that the economic benefits of education are not equally distributed across racial groups. For White individuals, education typically translates into stable employment and economic security, fostering improved health through access to resources and reduced stress. In contrast, Black individuals face systemic challenges, such as labor market discrimination and limited wealth-building opportunities, which weaken the protective effects of education on economic and health outcomes.

The MDRs framework is well-documented, showing that Black Americans receive lower returns from human capital and socioeconomic resources like education, income, and employment [27-38]. Prior research reveals that, despite similar educational attainment, Black individuals often experience higher rates of unemployment, lower wages, and limited upward mobility compared to White counterparts. These diminished returns are attributed to structural racism embedded within societal systems, including education, employment, and healthcare. For example, studies show that Black Americans are more likely to be employed in lower-wage, high-stress jobs and face higher rates of workplace discrimination, which erodes the positive impact of education on economic and health outcomes [27-38].

Structural inequalities, social stratification, and systemic discrimination are key mechanisms driving MDRs in Black populations [8]. Due to historical segregation and systemic racism, Black individuals are often concentrated in neighborhoods with limited access to higher-paying jobs, while White Americans tend to live closer to areas with better job opportunities. Black Americans face systemic barriers across multiple domains, including labor markets, housing, and healthcare, which reduce the effectiveness of educational and economic resources. Labor market discrimination limits access to high-paying jobs and increases the likelihood of employment in physically and psychologically taxing roles, even for highly educated Black individuals. Limited access to wealth-building resources, such as favorable mortgage rates and high-quality education, constrains Black Americans’ ability to accumulate wealth and achieve long-term economic stability. These systemic inequities collectively reduce the health benefits typically associated with educational attainment, as financial strain and stress continue to impact health despite higher education levels [1-3,5,44-49].

### 4.1. Implications

The findings of this study have significant implications for public policy and interventions aimed at addressing racial economic and health disparities. Policymakers should recognize that just equal access to resources, such as education, does not inherently lead to equal outcomes; to address MDRs, policies must go beyond universal approaches and focus on dismantling the structural barriers that prevent Black Americans from converting educational and economic achievements into meaningful health and economic benefits. Specifically, rigorous enforcement of anti-discrimination policies in education, employment, and housing is essential, along with initiatives that address wage gaps and promote job stability for Black Americans. Tailored interventions that address unique challenges facing Black communities, such as targeted employment programs, neighborhood investments, and improved access to affordable healthcare, could help mitigate the effects of MDRs and promote greater equity.

Addressing MDRs effectively requires multilevel policy responses that consider the interconnected nature of structural racism and economic disparities. Policies in health, social, and economic sectors must collectively address the myriad systems—education, housing, healthcare, and justice—that uphold racial inequities. By advocating for integrative policies that address the root causes of MDRs, such as systemic racism and socioeconomic stratification, policymakers can foster sustainable improvements in economic and health outcomes for Black Americans.

Addressing MDRs requires both equitable resource distribution and dismantling structural barriers that reduce the socioeconomic and health returns for Black Americans. Multidimensional solutions that address the complexity of MDRs and structural inequalities can pave the way for sustained reductions in racial disparities.

Policy solutions must move beyond merely equalizing access to resources such as education. Effective policies would also target discrimination in educational attainment and the labor market. Raising the minimum wage and reducing wage gaps can help bridge economic disparities, enhancing the economic security of low-wage earners, especially in occupations predominantly held by Black Americans. Income-based solutions like these could reinforce health and economic benefits that might otherwise be eroded by racial inequalities.

A core focus of MDRs is the elimination of discrimination across all levels. Enforcing anti-discrimination laws rigorously in housing, education, healthcare, and employment could reduce structural barriers that hinder Black Americans from translating socioeconomic gains into improved economic and health outcomes. Policies that promote neighborhood desegregation, ensure equitable educational opportunities, and reduce occupational hazards in jobs predominantly held by Black workers could increase the effectiveness of resources for Black Americans. Furthermore, investments in cultural competence and anti-bias training within workplaces, schools, and healthcare institutions can help reduce the effects of interpersonal discrimination that undermine economic and health gains.

There is not even equal access to resources such as education and income across racial and ethnic groups. Even if access were equalized, it would not necessarily lead to equal outcomes across these groups. Therefore, policies must go beyond access and address structural barriers that hinder Black Americans' ability to translate socioeconomic resources into meaningful economic and health gains. For example, policies could support tailored programs in predominantly Black neighborhoods and schools, reduce food deserts, and foster health-promoting environments. These targeted investments would help meet the specific needs of Black communities and dismantle the systemic barriers that limit the socioeconomic and health benefits of resources.

### 4.2. Leveraging Community and Employment Empowerment Programs

Employment empowerment programs within Black communities may also be beneficial, as these can provide resilience against adversity. Supporting community centers, skill-building organizations, and labor programs that already offer significant economic and health benefits could amplify their impact. Research shows that such programs yield substantial benefits for Black Americans, making them a culturally relevant and effective focus for intervention.

### 4.3. Limitations

This study has several limitations. First, the cross-sectional design limits our ability to establish causality. While our findings align with the MDRs framework, longitudinal studies are necessary to better understand how educational attainment impacts economic stability and health outcomes over time in Black and White populations. Additionally, this analysis was restricted to three specific income sources (Social Security disability income, disability benefits, and public assistance). Future studies could investigate a broader range of socioeconomic outcomes, including employment rates, occupational status, and wealth accumulation, to provide a more comprehensive understanding of MDRs. Furthermore, residual confounding may be present, as factors such as neighborhood context, social network access, and personal experiences of discrimination were not directly measured.

## 5. Conclusion

Our study highlights persistent disparities in the economic returns on educational attainment for Black Americans, supporting the MDRs framework and demonstrating that higher education levels do not offer equivalent protective effects against reliance on Social Security and disability income for Black adults compared to White adults. These findings emphasize the need for policy interventions that go beyond equal access to resources and actively address the structural barriers limiting the socioeconomic and health potential of Black Americans. Black communities need interventions that facilitate access to higher-paying jobs that can reduce reliance on Social Security and disability income. Addressing their health needs is equally essential to reduce the necessity of disability reliance. Through targeted, multilevel policies that tackle labor market inequities, housing discrimination, and healthcare disparities, policymakers can work toward a more equitable society where educational achievements lead to meaningful health and economic benefits for all racial groups.

## Figures and Tables

**Table 1.** Descriptives Overall and by Race

	All		White		Black	
	%	SE	%	SE	%	SE
Race						
White	83.50	0.57	-	-	-	-
Black	16.49	0.57	-	-	-	-
Education*						
Less than High School	9.44	0.30	8.51	0.30	14.16	0.89
High School Graduate	55.92	0.46	54.71	0.50	62.08	1.04
Bachelor or More	34.62	0.50	36.77	0.55	23.74	0.92
Marital Status*						
Other	50.29	0.45	46.56	0.47	69.17	1.03
Married	49.70	0.45	53.43	0.47	30.82	1.03
Last Week Employment*						
Unemployed	43.23	0.44	43.19	0.47	43.40	1.11
Employed	56.76	0.44	56.80	0.47	56.59	1.11
Gender*						
Female	50.67	0.40	50.12	0.42	53.46	1.04
Male	49.32	0.40	49.87	0.42	46.53	1.04
Social Security Income*						
No	69.63	0.42	68.55	0.45	75.07	0.96
Yes	30.36	0.42	31.44	0.45	24.92	0.96
Social Security Disability Income*						
No	90.52	0.27	91.47	0.28	85.68	0.83
Yes	9.47	0.27	8.52	0.28	14.31	0.83
Disability Benefit Income*						
No	91.88	0.25	92.70	0.25	87.73	0.81
Yes	8.11	0.25	7.29	0.25	12.26	0.81
Income From Public Assistance*						
No	96.89	0.17	97.38	0.16	94.43	0.62
Yes	3.10	0.17	2.61	0.16	5.56	0.62
	Mean	SE	Mean	SE	Mean	SE
Age (Years)*	49.81	.18	50.55	.19	46.08	.39

Data Source: National Center for Health Statistics, National Health Interview Survey, 2023.

* p < 0.05

**Table 2.** Summary of logistic regressions without interactions

	Odds ratio	Linearized Std. Err.	[95% conf.	interval]	p
Social Security Income					
Race (Black)	0.89	0.07	0.76	1.04	0.134
Age (Years)	1.09	0.00	1.09	1.10	< 0.001
Gender (Male)	0.99	0.05	0.90	1.08	0.757
Family Structure (Married)	0.82	0.04	0.75	0.90	< 0.001
Employment (Employed During Last Week)	0.36	0.02	0.32	0.39	< 0.001
Educational Attainment					
Less than High School Diploma					
High School Diploma	1.00	0.09	0.84	1.19	0.974
College Degree or More	0.66	0.06	0.55	0.78	< 0.001
Intercept	0.01	0.00	0.01	0.01	< 0.001
					
Social Security Disability Income					
Race (Black)	1.47	0.12	1.25	1.73	< 0.001
Age (Years)	1.00	0.00	0.99	1.00	0.026
Gender (Male)	1.19	0.07	1.05	1.34	0.005
Family Structure (Married)	0.78	0.05	0.68	0.88	< 0.001
Employment (Employed During Last Week)	0.33	0.02	0.28	0.38	< 0.001
Educational Attainment					
Less than High School Diploma					
High School Diploma	0.50	0.04	0.43	0.58	< 0.001
College Degree or More	0.17	0.02	0.14	0.21	< 0.001
Intercept	0.45	0.06	0.35	0.58	< 0.001
					
Disability Benefit Income					
Race (Black)	1.44	0.13	1.21	1.71	< 0.001
Age (Years)	0.99	0.00	0.99	1.00	< 0.001
Gender (Male)	1.22	0.08	1.08	1.39	0.002
Family Structure (Married)	0.85	0.06	0.74	0.96	0.012
Employment (Employed During Last Week)	0.31	0.02	0.26	0.36	< 0.001
Educational Attainment					
Less than High School Diploma					
High School Diploma	0.48	0.04	0.40	0.56	< 0.001
College Degree or More	0.14	0.02	0.12	0.18	< 0.001
Intercept	0.46	0.06	0.35	0.61	< 0.001
					
Income From Public Assistance					
Race (Black)	1.67	0.23	1.28	2.19	< 0.001
Age (Years)	0.98	0.00	0.98	0.99	< 0.001
Gender (Male)	0.64	0.06	0.53	0.78	< 0.001
Family Structure (Married)	0.71	0.09	0.55	0.91	0.007
Employment (Employed During Last Week)	0.57	0.06	0.46	0.71	< 0.001
Educational Attainment					
Less than High School Diploma					
High School Diploma	0.67	0.09	0.51	0.88	0.004
College Degree or More	0.25	0.04	0.18	0.35	< 0.001
Intercept	0.21	0.04	0.14	0.31	< 0.001

Data Source: National Center for Health Statistics, National Health Interview Survey, 2023.

**Table 3.** Summary of logistic regressions with interactions

	Odds ratio	Linearized Std. Err.	[95% conf.	interval]	p
Social Security Income					
Race (Black)	0.55	0.11	0.37	0.80	0.002
Age (Years)	1.09	0.00	1.09	1.10	< 0.001
Gender (Male)	0.99	0.05	0.90	1.08	0.761
Family Structure (Married)	0.82	0.04	0.75	0.90	< 0.001
Employment (Employed During Last Week)	0.36	0.02	0.32	0.39	< 0.001
Educational Attainment					
Less than High School Diploma					
High School Diploma	0.87	0.09	0.72	1.06	0.179
College Degree or More	0.58	0.06	0.47	0.70	< 0.001
Education x Race					
High School Diploma x Race	1.79	0.39	1.16	2.74	0.008
College Degree or More x Race	1.75	0.42	1.09	2.81	0.021
Intercept	0.01	0.00	0.01	0.01	< 0.001
					
Social Security Disability Income					
Race (Black)	1.27	0.19	0.95	1.71	0.112
Age (Years)	1.00	0.00	0.99	1.00	0.028
Gender (Male)	1.19	0.07	1.06	1.34	0.004
Family Structure (Married)	0.78	0.05	0.69	0.88	< 0.001
Employment (Employed During Last Week)	0.33	0.02	0.28	0.38	< 0.001
Educational Attainment					
Less than High School Diploma					
High School Diploma	0.48	0.04	0.40	0.57	< 0.001
College Degree or More	0.15	0.02	0.12	0.19	< 0.001
Education x Race					
High School Diploma x Race	1.15	0.21	0.81	1.64	0.429
College Degree or More x Race	1.64	0.42	0.99	2.70	0.053
Intercept	0.47	0.06	0.36	0.60	< 0.001
					
Disability Benefit Income					
Race (Black)	1.26	0.20	0.92	1.73	0.147
Age (Years)	0.99	0.00	0.99	1.00	< 0.001
Gender (Male)	1.23	0.08	1.08	1.39	0.002
Family Structure (Married)	0.85	0.06	0.74	0.97	0.014
Employment (Employed During Last Week)	0.31	0.02	0.26	0.36	< 0.001
Educational Attainment					
Less than High School Diploma					
High School Diploma	0.46	0.04	0.38	0.55	< 0.001
College Degree or More	0.13	0.02	0.11	0.17	< 0.001
Education x Race					
High School Diploma x Race	1.16	0.22	0.80	1.69	0.427
College Degree or More x Race	1.40	0.43	0.76	2.55	0.279
Intercept	0.48	0.07	0.36	0.63	< 0.001
					
Income From Public Assistance					
Race (Black)	1.28	0.34	0.76	2.15	0.360
Age (Years)	0.98	0.00	0.98	0.99	< 0.001
Gender (Male)	0.64	0.06	0.53	0.78	< 0.001
Family Structure (Married)	0.72	0.09	0.56	0.92	0.009
Employment (Employed During Last Week)	0.57	0.06	0.46	0.70	< 0.001
Educational Attainment					
Less than High School Diploma					
High School Diploma	0.62	0.10	0.46	0.84	0.002
College Degree or More	0.19	0.04	0.13	0.28	< 0.001
Education x Race					
High School Diploma x Race	1.24	0.37	0.69	2.22	0.467
College Degree or More x Race	2.69	1.03	1.27	5.70	0.010
Intercept	0.23	0.05	0.15	0.34	< 0.001

Data Source: National Center for Health Statistics, National Health Interview Survey, 2023.
